# On neglecting Coriolis and related couplings in first-principles rovibrational spectroscopy: considerations of symmetry, accuracy, and simplicity

**DOI:** 10.1038/s41598-020-60971-x

**Published:** 2020-03-17

**Authors:** János Sarka, Bill Poirier, Viktor Szalay, Attila G. Császár

**Affiliations:** 1 Department of Chemistry and Biochemistry, Texas Tech University, Lubbock, Texas 79409 USA; 2 Institute for Solid State Physics and Optics, Wigner Research Centre for Physics, P.O. Box 49, H-1525 Budapest, Hungary; 3 MTA-ELTE Complex Chemical Systems Research Group and Laboratory of Molecular Structure and Dynamics, Institute of Chemistry, ELTE Eötvös Loránd University, H-1117 Budapest, Pázmány Péter sétány 1/A Hungary

**Keywords:** Chemistry, Theoretical chemistry, Method development

## Abstract

The rotation-vibration (Coriolis) coupling contribution to variationally computed rovibrational energy levels is investigated, employing triatomic AB$${}_{2}$$ molecules as models. In particular, calculations are performed for H$${}_{2}$$$${}^{16}$$O, across a range of vibrational and rotational excitations, both with and without the Coriolis contribution. A variety of different embedding choices are considered, together with a hierarchy of increasingly severe approximations culminating in a generalized version of the so-called “centrifugal sudden” method. Several surprising and remarkable conclusions are found, including that the Eckart embedding is *not* the best embedding choice.

## Introduction

Exact rotation-vibration Hamiltonians always have the form

1 $${ {\hat{H}} }_{{\rm{V}}{\rm{R}}}=\widehat{T}+\widehat{V}={\widehat{T}}_{{\rm{V}}}+{\widehat{T}}_{{\rm{R}}}+{\widehat{T}}_{{\rm{V}}{\rm{R}}}+\widehat{V},$$

where the vibration-rotation (VR) coupling contribution to the kinetic energy operator (KEO) $\widehat{T}$^1,2^—*i.e*., ${\widehat{T}}_{{\rm{V}}{\rm{R}}}$—is often called the “Coriolis coupling” (CC) term. Since variational computation^3–19^ of rovibrational energy levels utilizing ${ {\hat{H}} }_{{\rm{V}}{\rm{R}}}$ are expensive, both in terms of memory and CPU time, it is highly useful that the rotational quantum number $J$ is a good quantum number^20^ and thus the matrix representation of ${ {\hat{H}} }_{{\rm{V}}{\rm{R}}}$ is block-diagonal in $J$. Then it is natural to consider KEO approximations that separate rotational and vibrational motions within these blocks in some form. Ideally, such approximations would apply to molecules with complicated internal motion^21–29^, as well as to semirigid ones. Hereby we explore in a joint analytical and numerical treatment—for the first time to our knowledge—the impact on accuracy and numerical efficiency that results when ${\widehat{T}}_{{\rm{V}}{\rm{R}}}$ is neglected, employing different vibrational coordinates and different embeddings of the molecule-fixed axes. Such a study has been called the “logical next step” needed to confirm or refute theoretical hypotheses put forth in previous work^30^. Additionally, we examine what happens when parts of ${\widehat{T}}_{{\rm{R}}}$ are also discarded. Since $\widehat{V}$ depends only on vibrational coordinates, one may imagine that neglecting ${\widehat{T}}_{{\rm{V}}{\rm{R}}}$ from Eq. (1) will “decouple” the rotational-vibrational problem, thereby leading, *e.g*., to a significant reduction in computational effort. As it turns out (see below), further approximations must be applied to realize such savings. Finally, we introduce a natural hierarchy of approximations, culminating in a generalized version of the centrifugal sudden (CS) method^31–39^, for which our analysis provides a nice theoretical framework.

In this paper special attention is given to the choice of embedding in first-principles variational rovibrational computations^40^. If the full $${ {\hat{H}} }_{{\rm{V}}{\rm{R}}}$$ is used, converged numerical rovibrational energy levels should be independent of the choice of the vibrational coordinates and the embedding. This is, however, *not* the case when $${\widehat{T}}_{{\rm{V}}{\rm{R}}}$$ is neglected, since $${\widehat{T}}_{{\rm{V}}{\rm{R}}}$$ itself depends on the embedding (although not on the vibrational coordinates, as is clear from abstract operator notation). Some embeddings are therefore better than others, in terms of having smaller deviations between the exact and the approximate computed energies. Note that it is impossible to define a frame in which the Coriolis coupling vanishes over the whole configuration space^40^. We therefore explore the role of embedding on the magnitude of the energy contributions related to the Coriolis term at every rung of the approximation hierarchy. We restrict consideration to triatomic AB$${}_{2}$$ molecules, using H$${}_{2}$$$${}^{16}$$O as our canonical test case. Furthermore, a subset of just three, “linear” embeddings is considered here, as these have been found to perform best in our numerical tests^41^.

The present study builds on previous theoretical work on rotational and vibrational coordinate separation^40,42–44^ and coupling^2,30,45,46^. In particular, Sutcliffe and Tennyson derived general rovibrational Hamiltonians in terms of axis embeddings for triatomic molecules^47,48^. Mardis and Sibert^49^ derived a Casimir-bond operator, whereby the CC term is zero at equilibrium. Wei and Carrington^50^ investigated Eckart embeddings^51,52^—and their bond and bisector counterparts—for triatomics using Radau, valence, and Jacobi vibrational coordinates. The same authors derived triatomic Eckart-embedded Hamiltonian operators for valence^30^ and Radau^53^ coordinates. Wei and Carrington^30^ investigated the properties of both operators, and compared the Coriolis coupling in the different operators focusing on the ${{\bf{G}}}_{{\rm{V}}{\rm{R}}}$ tensor elements. They concluded that Eckart embedding is the best choice when ${\widehat{T}}_{{\rm{V}}{\rm{R}}}$ is neglected^53^, a basic assumption, which has never since been questioned. Furthermore, they also claim^30^ that although they made the first step at discussing the different operators’ relative advantages, to really compare their efficacy it is necessary to calculate ro-vibrational energy levels^30^, a task performed during this study. In 1974, McGuire and Kouri developed the “${j}_{z}$ conserving” centrifugal sudden approximation in a Jacobi coordinate framework, for three-atom (atom+diatom) quantum scattering calculations^32^; in the same year, Pack published his paper on related “sudden approximation” methods^31^, specifically comparing space-fixed and body-fixed formulations. The present study generalizes the centrifugal sudden approximation.

## Triatomic AB${}_{2}$ molecules: coordinates and embeddings

**Figure 1:**
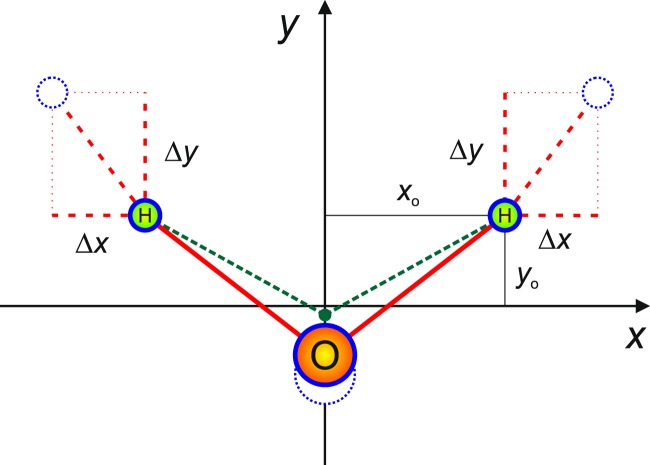
The symmetric vibrational displacement coordinates, $\Delta x$ and $\Delta y$, used to define and compare the three linear embeddings investigated in this work. For all such embeddings, and for all pure symmetric displacements from the reference equilibrium geometry ($\Delta x=\Delta y=0$), the displaced geometries exhibit ${C}_{2v}$ point-group symmetry, with the body-fixed $x$ axis corresponding to the H–H separation vector, the $y$ axis to the angle bisector, and the $z$ axis to the normal to the molecular plane.

Consider a triatomic AB${}_{2}$ molecule with a reference geometry of ${C}_{2v}$ point-group symmetry (Fig. 1). Upon removing the center-of-mass motion, six independent coordinates remain. Three of these are vibrational coordinates, so that in effect there is a (local) three-parameter family of possible embedding choices. Furthermore, triatomic systems are always planar (except for collinear geometries), effectively reducing the range of local embedding choices to just a one-parameter family.

**Figure 2:**
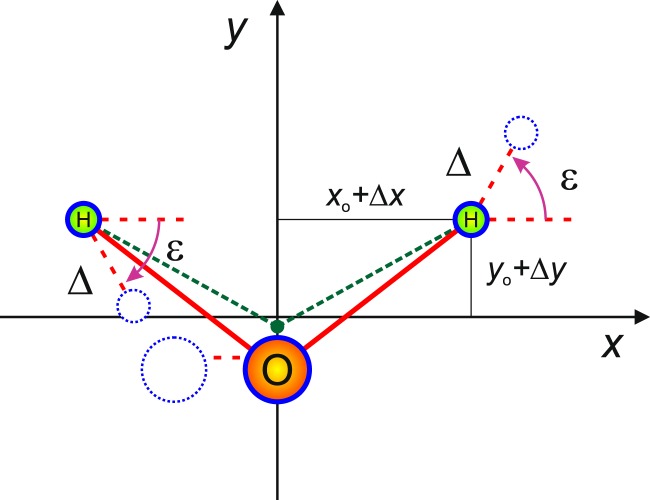
The asymmetric vibrational coordinate, $\Delta$, and the embedding angle, $\varepsilon$, used to define and compare the three linear embeddings of this work. A displacement $\Delta$ gives rise to asymmetric ${C}_{s}$ geometries. The body-fixed $z$ axis is normal to the molecular plane, the $x$ and $y$ axes vary with the embedding, depending on the value of the embedding angle, $\varepsilon$. It is often convenient to replace the asymmetric displacement parameter $\Delta$ with $\mu =\Delta {\rm{\cos }}\,\varepsilon$.

All linear embeddings behave the same with respect to symmetric vibrational displacements. We thus take $$\Delta x$$ and $$\Delta y$$ of Fig. 1 as the two symmetric vibrational coordinates, describing symmetric stretch and bend motions. Additionally, for all linear embeddings, the pure asymmetric stretch vibrational coordinate corresponds to a linear displacement by the distance $$\Delta $$, in a direction for B$${}_{1}$$/B$${}_{2}$$ (H$${}_{1}$$/H$${}_{2}$$ in Figs. 1 and 2, reflecting the fact that our AB$${}_{2}$$ test molecule is H$${}_{2}$$$${}^{16}$$O) that is at an angle $$\varepsilon $$ below/above the $$\widehat{x}$$ axis (see Fig. 2). Different linear embeddings therefore differ only with respect to the value of the angle $$\varepsilon $$. Only three of the many possible $$\varepsilon $$ choices are considered here: Eckart embedding (EE), “Radau bisector” embedding (RBE), and “valence bisector” embedding (VBE), defined as products of a mass factor and a geometry factor:

2 $$\tan \ {\varepsilon }^{{\rm{E}}{\rm{E}}}=\left(1+\left[\frac{2m}{M}\right]\right)\left(\frac{{y}_{0}}{{x}_{0}}\right)$$

3 $$\tan \ {\varepsilon }^{{\rm{R}}{\rm{B}}{\rm{E}}}=\left(1+\left[\frac{2m}{M}\right]\right)\left(\frac{{y}_{0}+\Delta y}{{x}_{0}+\Delta x}\right)$$

4 $$\tan \ {\varepsilon }^{{\rm{V}}{\rm{B}}{\rm{E}}}={\left(1+\left[\frac{2m}{M}\right]\right)}^{2}\left(\frac{{y}_{0}+\Delta y}{{x}_{0}+\Delta x}\right)$$

In many respects, RBE lies “halfway” between EE and VBE. In particular, Eq. (3) becomes equal to Eq. (4) in the $$(m/M)\to 0$$ limit (for the H$${}_{2}$$$${}^{16}$$O molecule considered here, $$M$$ is the nuclear mass of H, $$m$$ = 1.007 276 47 u, while $$M$$ is the nuclear mass of $${}^{16}$$O, $$M$$ = 15.990 526 00 u). This is true regardless of $$\Delta x$$ and $$\Delta y$$, because the geometric (*i.e*., second) factors in the right hand side of the two equations are identical. It is the mass (*i.e*., the first) factor that is identical in Eqs. (2) and (3), whereas the RBE geometric factor has been modified from the EE form to include the symmetric displacements. Note that pure asymmetric stretching is the same for both EE and RBE, since this corresponds to $$\Delta x=\Delta y=0$$. Furthermore, in the vicinity of the reference geometry, EE and RBE are locally equivalent to each other, but different from VBE. These characteristics have important repercussions, as discussed below.

Note that in order to qualitatively compare the three linear embeddings chosen, it is advantageous to choose the same set of vibrational coordinates ($\Delta x$, $\Delta y$, and $\Delta$). This is done in this section and the next. Nevertheless, the actual first-principles numerical computations of this study were carried out using valence and Radau internal coordinates (see below). As discussed, the final computed eigenvalues—whether with or without ${\widehat{T}}_{{\rm{V}}{\rm{R}}}$— *should not depend* on the choice of vibrational coordinates, although this statement will be tested explicitly.

## Classical and quantum Hamiltonians & approximations

The three contributions to the rovibrational KEO of Eq. (1) come from the blocks of the well-known covariant and contravariant ${\bf{G}}$ and ${\bf{g}}$ tensors of nuclear-motion theory^54,55^

5 $${\bf{G}}=\left(\begin{array}{cc}{{\bf{G}}}_{{\rm{V}}} & {{\bf{G}}}_{{\rm{V}}{\rm{R}}}\\ {{\bf{G}}}_{{\rm{V}}{\rm{R}}}^{T} & {{\bf{G}}}_{{\rm{R}}}\end{array}\right)={{\bf{g}}}^{-1}={\left(\begin{array}{cc}{{\bf{g}}}_{{\rm{V}}} & {{\bf{g}}}_{{\rm{V}}{\rm{R}}}\\ {{\bf{g}}}_{{\rm{V}}{\rm{R}}}^{T} & {{\bf{g}}}_{{\rm{R}}}\end{array}\right)}^{-1},$$

where each block in Eq. (5) is $3\times 3$. For certain geometries and embeddings, it may be the case that the VR coupling, ${{\bf{g}}}_{{\rm{V}}{\rm{R}}}$, vanishes. In such cases ${\bf{g}}$ becomes block diagonal, so that:

6 $${{\bf{G}}}_{{\rm{V}}}={{\bf{g}}}_{{\rm{V}}}^{-1};\ {{\bf{G}}}_{{\rm{V}}{\rm{R}}}=0;\ {{\bf{G}}}_{{\rm{R}}}={{\bf{g}}}_{{\rm{R}}}^{-1}.$$

Thus, ${{\bf{G}}}_{{\rm{V}}{\rm{R}}}$ also vanishes and ${{\bf{G}}}_{{\rm{R}}}$ is “geometric”—meaning simply that it is the inverse of the moment-of-inertia tensor, ${{\bf{g}}}_{{\rm{R}}}$. More generally—*i.e*., when ${{\bf{g}}}_{{\rm{V}}{\rm{R}}}$ and ${{\bf{G}}}_{{\rm{V}}{\rm{R}}}$ are not zero—the VR coupling modifies the form of ${{\bf{G}}}_{{\rm{R}}}$ as follows:

7 $${{\bf{G}}}_{{\rm{R}}}={({{\bf{g}}}_{{\rm{R}}}-{{\bf{g}}}_{{\rm{V}}{\rm{R}}}^{T}\cdot {{\bf{g}}}_{{\rm{V}}}^{-1}\cdot {{\bf{g}}}_{{\rm{V}}{\rm{R}}})}^{-1}.$$

Thus, the three rotational constants, defined as the eigenvalues of ${{\bf{G}}}_{{\rm{R}}}$, are no longer equal to the inverses of the three moments of inertia^56,57^.

Note that ${{\bf{G}}}_{{\rm{R}}}$ as defined by Eq. (7), and also ${{\bf{g}}}_{{\rm{R}}}^{-1}$, are manifestly independent of the choice of vibrational coordinates. This is *not* true of the tensor components of ${{\bf{G}}}_{{\rm{V}}{\rm{R}}}$, which therefore serve as *unreliable* indicators of the true magnitude of the VR coupling, despite the fact that they have been used in this way in the past^30,53^. Instead, we propose using the tensor $({{\bf{G}}}_{{\rm{R}}}-{{\bf{g}}}_{{\rm{R}}}^{-1})$ for this purpose. Not only is this tensor vibrational-coordinate-independent, but its Frobenius norm, $| | {{\bf{G}}}_{{\rm{R}}}-{{\bf{g}}}_{{\rm{R}}}^{-1}| {| }_{{\rm{F}}}$, is also independent of rotations.

The structure of triatomic molecules remains always planar, leading to a block-diagonal planar ($xy$) and perpendicular ($z$) structure for ${{\bf{g}}}_{{\rm{R}}}$ and ${{\bf{G}}}_{{\rm{R}}}$:

8 $${{\bf{g}}}_{{\rm{R}}}=\left(\begin{array}{ccc}{g}_{xx}^{{\rm{R}}} & {g}_{xy}^{{\rm{R}}} & 0\\ {g}_{yx}^{{\rm{R}}} & {g}_{yy}^{{\rm{R}}} & 0\\ 0 & 0 & {g}_{zz}^{{\rm{R}}}\end{array}\right);\ {{\bf{G}}}_{{\rm{R}}}=\left(\begin{array}{ccc}{G}_{xx}^{{\rm{R}}} & {G}_{xy}^{{\rm{R}}} & 0\\ {G}_{yx}^{{\rm{R}}} & {G}_{yy}^{{\rm{R}}} & 0\\ 0 & 0 & {G}_{zz}^{{\rm{R}}}\end{array}\right).$$

Regardless of the embedding, ${g}_{zz}^{{\rm{R}}}$ is always equal to the perpendicular moment of inertia, ${I}_{z}={I}_{x}+{I}_{y}$. In contrast, the individual ${{\bf{g}}}_{xy}$ tensor elements vary with the embedding, although the two eigenvalues of ${{\bf{g}}}_{xy}$ are always equal to the two planar moments, ${I}_{x}$ and ${I}_{y}$.

As to ${{\bf{G}}}_{{\rm{R}}}$, there is more variability, since this tensor is in general not geometric. Nevertheless, it can be shown that for *all* geometries and embeddings the ${{\bf{G}}}_{xy}$ block is *always* geometric—meaning that ${{\bf{G}}}_{xy}={{\bf{g}}}_{xy}^{-1}$, and the two planar rotational constants are always ${A}_{x}=1/{I}_{x}$ and ${A}_{y}=1/{I}_{y}$. Moreover, for all ${C}_{2v}$ geometries and linear embeddings, the off-diagonal ${G}_{xy}^{{\rm{R}}}$ tensor element vanishes, so that ${A}_{x}={G}_{xx}^{{\rm{R}}}$ and ${A}_{y}={G}_{yy}^{{\rm{R}}}$. Thus, the *only* place where the non-geometric character of ${{\bf{G}}}_{{\rm{R}}}$ can manifest is in the third rotational constant, ${A}_{z}={G}_{zz}^{{\rm{R}}}$^56^; in general, ${A}_{z}={G}_{zz}^{{\rm{R}}}\ne 1/{I}_{z}=1/{g}_{zz}^{{\rm{R}}}$. The difference, $({G}_{zz}^{{\rm{R}}}-1/{g}_{zz}^{{\rm{R}}})$, comes about because of non-zero Coriolis coupling. It can be shown that *only* the $z$ components of the Coriolis coupling—*i.e*., the ${G}_{iz}^{{\rm{V}}{\rm{R}}}$ tensor elements, where $i$ is the index of the vibrational coordinates—are non-zero. Note that ${A}_{z}$ need not in general be the smallest rotational constant, though certainly it is smallest in the geometric case.

For all linear embeddings, ${{\bf{G}}}_{{\rm{R}}}$ has the general form 9Note that ${G}_{xx}^{{\rm{R}}}={A}_{x}$, ${G}_{yy}^{{\rm{R}}}={A}_{y}$, and ${G}_{xy}^{{\rm{R}}}=0$ for all ${C}_{2v}$ geometries (whereby ${\mu }=0$), as predicted. However, even for ${C}_{2v}$ geometries, ${G}_{zz}^{{\rm{R}}}$ depends on the embedding. Specific forms may be obtained by substituting $\tan \varepsilon$ to Eq. (9) via Eqs. (2), (3), or (4), as appropriate. Doing so reveals something quite special about the RBE—namely, ${G}_{zz}^{{\rm{R}}}={A}_{z}=1/{I}_{z}$ when $\Delta =0$, so that ${{\bf{G}}}_{{\rm{R}}}$ is geometric in this case (see Table 1 for numerical examples). Thus, we arrive at the first important conclusion of this study, namely that *RBE is the only linear embedding for which Coriolis coupling vanishes for all* ${C}_{2v}$ *geometries*. This property is in principle discernible from the form of the operator as derived in Eqs. (3)–(19) of ref. ^53^; however, it appears not to have been noticed previously. Indeed, ref. ^53^ even claims that the Eckart embedding is superior to RBE. For general molecules, it is well known that Coriolis coupling in the Eckart embedding always vanishes at least at one point, *i.e*., at the reference geometry. In the special case of AB${}_{2}$ molecules, however, Coriolis coupling vanishes across a one-parameter family of geometries, defined by the pure symmetric stretch motion^53^. On the other hand, the VBE ${{\bf{G}}}_{{\rm{R}}}$ is not geometric—and so Coriolis coupling does not vanish—even at the reference geometry itself.

**Table 1:** Numerical values of the $${{\bf{G}}}_{{\rm{R}}}$$ and $${{\bf{G}}}_{{\rm{V}}{\rm{R}}}$$ tensor elements for all of the embeddings studied in this paper. The only non-zero elements, the $$z$$ components of the Coriolis coupling—*i.e*., the $${G}_{iz}^{{\rm{V}}{\rm{R}}}$$ tensor elements—are shown here, in cm$${}^{-1}$$. The numbers provided correspond to the $${C}_{2v}$$ reference equilibrium structure of H$${}_{2}$$$${}^{16}$$O, $${r}_{e}$$ = 0.957 820 Å and $${\theta }_{e}$$ = 104.500$${}^{\circ }$$ in valence coordinates, and several symmetrically and asymmetrically distorted geometries with either $${C}_{2v}$$ or $${C}_{s}$$ point-group symmetry. The notation “$${C}_{2v}$$ ($$x$$, $$y$$)/$${C}_{s}$$ ($$x$$, $$y$$)” refers to a symmetric/asymmetric stretch distortion of $$x$$ % and a bend distortion of $$y$$ % relative to the reference structure. FNGR is the Frobenius norm ($$| | {{\bf{G}}}_{{\rm{R}}}-{{\bf{g}}}_{{\rm{R}}}^{-1}| {| }_{{\rm{F}}}$$) of the $$({{\bf{G}}}_{{\rm{R}}}-{{\bf{g}}}_{{\rm{R}}}^{-1})$$ tensor, where $${{\bf{G}}}_{{\rm{R}}}$$ and $${{\bf{g}}}_{{\rm{R}}}$$ are defined in Eq. (8).

Embedding	Symmetry	**G**${}_{{\bf{R}}}$				FNGR	**G**${}_{{\bf{V}}{\bf{R}}}$		
		${{\boldsymbol{G}}}_{{\bf{x}}{\bf{x}}}$	${{\boldsymbol{G}}}_{{\boldsymbol{x}}{\boldsymbol{y}}}$	${{\boldsymbol{G}}}_{{\boldsymbol{y}}{\boldsymbol{y}}}$	${{\boldsymbol{G}}}_{{\boldsymbol{z}}{\boldsymbol{z}}}$		${{\boldsymbol{G}}}_{{\boldsymbol{1}}{\boldsymbol{z}}}$	${{\boldsymbol{G}}}_{{\boldsymbol{2}}{\boldsymbol{z}}}$	${{\boldsymbol{G}}}_{{\boldsymbol{3}}{\boldsymbol{z}}}$
(valence) Eckart	${C}_{2v}$ (ref.)	54.80	0	29.18	19.04	0	0	0	0
(Radau) Eckart	${C}_{2v}$ (ref.)	54.80	0	29.18	19.04	0	0	0	0
valence bisector	${C}_{2v}$ (ref.)	54.80	0	29.18	19.10	0.06	2.01	-2.01	0
Radau bisector	${C}_{2v}$ (ref.)	54.80	0	29.18	19.04	0	0	0	0
(valence) Eckart	${C}_{2v}$ (10, 0)	45.29	0	24.11	15.74	0	0	0	0
(Radau) Eckart	${C}_{2v}$ (10, 0)	45.29	0	24.11	15.74	0	0	0	0
valence bisector	${C}_{2v}$ (10, 0)	45.29	0	24.11	15.79	0.05	1.83	-1.83	0
Radau bisector	${C}_{2v}$ (10, 0)	45.29	0	24.11	15.74	0	0	0	0
(valence) Eckart	${C}_{2v}$ (10, 30)	120.19	0	17.56	16.45	1.13	8.65	-8.65	0
(Radau) Eckart	${C}_{2v}$ (10, 30)	120.19	0	17.56	16.45	1.13	8.22	-8.22	0
valence bisector	${C}_{2v}$ (10, 30)	120.19	0	17.56	15.34	0.03	1.32	-1.32	0
Radau bisector	${C}_{2v}$ (10, 30)	120.19	0	17.56	15.32	0	0	0	0
(valence) Eckart	${C}_{s}$ (10, 0)	56.50	-8.03	30.01	19.04	0.18	0.19	0.19	3.72
(Radau) Eckart	${C}_{s}$ (10, 0)	56.50	-8.03	30.01	19.04	0.18	0.02	-0.02	3.60
valence bisector	${C}_{s}$ (10, 0)	56.41	-8.17	30.11	19.69	0.83	2.24	-1.83	7.66
Radau bisector	${C}_{s}$ (10, 0)	56.50	-8.02	30.01	19.60	0.74	0	0	7.27
(valence) Eckart	${C}_{s}$ (10, 50)	518.14	-41.10	22.20	22.32	4.16	15.00	-17.83	1.17
(Radau) Eckart	${C}_{s}$ (10, 50)	518.14	-41.10	22.20	22.32	4.16	14.04	-16.98	1.02
valence bisector	${C}_{s}$ (10, 50)	520.73	-20.05	19.62	18.92	0.75	0.91	-0.75	3.12
Radau bisector	${C}_{s}$ (10, 50)	520.81	-18.93	19.53	18.83	0.66	0	0	2.64

Having discussed the structure of tensor ${\bf{G}}$, the next task is to construct the corresponding Hamiltonian operator and its matrix representation. The choice of vibrational basis set/vibrational coordinates is independent of the embedding and need not be considered further, except for verification purposes. For the rotational space, the usual^58^ Wigner rotation function basis, $\left|JKM\right\rangle$, can be used. This requires specification of the body-fixed axes $(\widehat{a},\widehat{b},{\hat{c}})$ (with ${\hat{c}}$ associated with $K$) in addition to the embedding itself. For all linear embeddings, and all exact and approximate Hamiltonians considered here, the rotational quantum numbers $J$ and $M$ are both rigorously good, but there is in general coupling with respect to $K$, giving rise to a $K$-block-pentadiagonal structure (*i.e*., non-zero matrix elements correspond to $| K-{K}^{{\prime} }| \le 2$).

The overall parity, $p=\pm 1$, is also a good quantum number. Through symmetry adaptation, the corresponding $M=0$ Hamiltonian matrix, ${\widetilde{H}}^{J}$, decouples into positive- and negative-parity symmetry blocks (replacing $-J\le K\le J$ with $0\le \bar{K}\le J$), thereby effectively reducing the basis size by a factor of two. In general, an $n$-fold reduction of the basis gives rise to a computational saving of ${n}^{2}$. AB${}_{2}$ systems are also characterized by permutation symmetry, $\varepsilon =\pm 1$, associated with B${}_{1}$–B${}_{2}$ exchange; this leads to a further factor-of-two reduction (*i.e*., $n=4$), with respect to even- and odd-$\bar{K}$ values.

The KEO approximations considered introduce additional Hamiltonian symmetries, which increase with the severity of the approximation^59^. The sequence of approximations is as follows. The *Coriolis-free* approximation (CFA) is the result of neglecting ${{\bf{G}}}_{{\rm{V}}{\rm{R}}}$. The CFA Hamiltonian then becomes a sum of pure vibration and pure rotation contributions, $({\widehat{T}}_{{\rm{V}}}+V)+{\widehat{T}}_{{\rm{R}}}$—although this Hamiltonian is still *not* separable, because ${\widehat{T}}_{{\rm{R}}}$ depends parametrically on the vibrational coordinates. If, in addition, we set ${G}_{xy}^{{\rm{R}}}=0$, we obtain the *diagonal* ${{\bf{G}}}_{{\rm{R}}}$ approximation (DGRA). This introduces a new permutation symmetry—together with an *almost* good quantum number that serves as an excellent state label. Finally, the *generalized CS* approximation (GCSA) is obtained by discarding all remaining $K$ coupling in the $\left|JKM\right\rangle$ representation. As a consequence, $K$ now also becomes a “good” quantum number. Note that the centrifugal sudden approximation has been widely used in the quantum dynamics field^31–39^, in the context of Jacobi and Radau coordinates and related embeddings^60^. To the best of our knowledge, we are the first to generalize the centrifugal sudden approximation for arbitrary embeddings.

## Results and Discussion

Numerical determination of the rovibrational energy levels of H$${}_{2}$$$${}^{16}$$O was based on the BT2 potential energy surface (PES)^61^ of H$${}_{2}$$$${}^{16}$$O and the GENIUSH code^3,18,19,62,63^—for the exact KEO as well as all three approximations described above, across a wide range of embeddings and rotational and vibrational excitations. In what follows, we focus only on the three linear embeddings, on $$J=1$$ and $$J=10$$, and on the lowest few vibrational parent states. For all Eckart embedding calculations, results were computed using both valence and Radau internal coordinates. In all such cases, the results were found to be identical, as expected.

### The Coriolis-free approximation (CFA)

The Coriolis-free approximation does *not* lead to new symmetries; thus, it provides no significant numerical advantages over exact calculations, although it may be useful for state labeling. On the other hand, being the least severe approximation, CFA is expected to be the most accurate.

In the Eckart embedding, ${{\bf{G}}}_{{\rm{V}}{\rm{R}}}={\bf{0}}$ for the reference geometry (usually taken as the global minimum of the PES); thus, we expect the greatest accuracy for the pure rotational states, *i.e*., those corresponding to the vibrational ground state, (${v}_{1}\ {v}_{2}\ {v}_{3}$) = (0 0 0), employing the canonical ordering of the vibrations. For VBE, in contrast, ${{\bf{G}}}_{{\rm{V}}{\rm{R}}}\ne {\bf{0}}$, even at the reference geometry. Nevertheless, chemical intuition suggests the importance of the VBE picture, and the B–A–B bisector is certainly relevant given the identical B atoms. This suggests that VBE will be less accurate than EE for the lowest-lying energy levels, but may provide greater accuracy further up in the spectrum, particularly for symmetric vibrational coordinate excitations.

**Figure 3:**
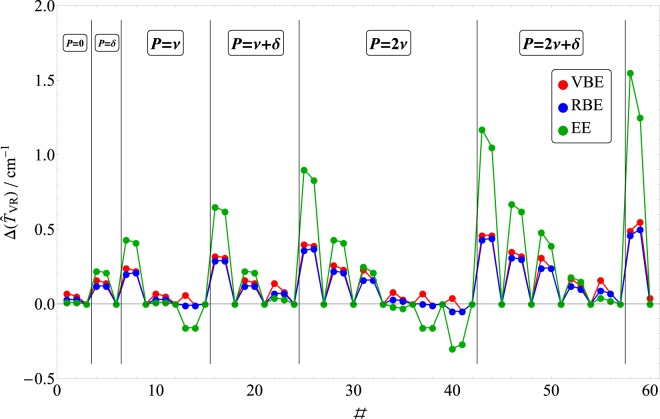
The differences, $\Delta ({\widehat{T}}_{{\rm{V}}{\rm{R}}})$, of the $J=1$ rovibrational energy levels of H${}_{2}$^16^O using the exact Hamiltonian, ${\hat{H}}$, and the Coriolis-free Hamiltonian, ${\hat{H}} -{\widehat{T}}_{{\rm{V}}{\rm{R}}}$. The color-coded results correspond to valence bisector (VBE), Radau bisector (RBE), and Eckart (EE) embeddings. The vibrational (${v}_{1}\ {v}_{2}\ {v}_{3}$) quantum numbers are assigned for each state and they are presented in the form of the resonance polyads, $n\nu$ or $n\nu +\delta$ according to the number of stretching ($\nu$) and bending ($\delta$) quanta, where two bending is “equivalent” to one stretching excitation.

**Figure 4:**
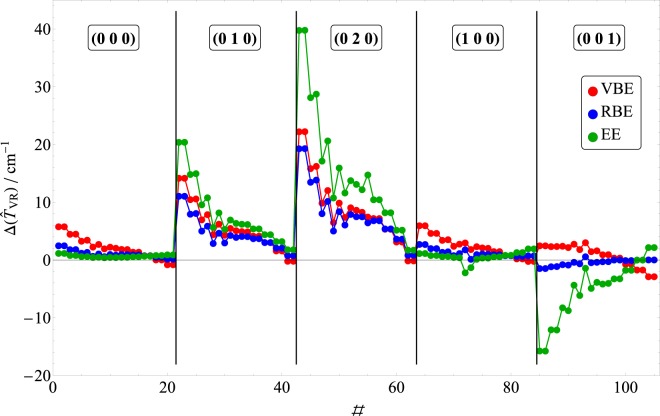
The differences, $\Delta ({\widehat{T}}_{{\rm{V}}{\rm{R}}})$, of the $J=10$ rovibrational energy levels of H${}_{2}$^16^O using the exact Hamiltonian, ${\hat{H}}$, and the Coriolis-free Hamiltonian, ${\hat{H}} -{\widehat{T}}_{{\rm{V}}{\rm{R}}}$. The color-coded results correspond to valence bisector (VBE), Radau bisector (RBE), and Eckart (EE) embeddings. Vibrational (${v}_{1}\ {v}_{2}\ {v}_{3}$) quantum numbers are assigned for each state.

The numerical results on H$${}_{2}$$$${}^{16}$$O bear out all of these predictions. We see from Table 1 that the EE $${{\bf{G}}}_{{\rm{V}}{\rm{R}}}$$ tensor elements increase from zero to 8.7 cm$${}^{-1}$$ under a symmetric vibrational coordinate displacement, whereas the corresponding VBE values vary over a narrower range, 0.7–1.7 cm$${}^{-1}$$. Likewise, discrepancies in the computed CFA *rovibrational* energies (see Table 2 and Fig. 3 for $$J=1$$ and Table 3 and Fig. 4 for $$J=10$$) show a highly marked increase with vibrational excitation that is more pronounced for EE than for VBE. On the other hand, $$J=1$$ EE CFA errors for (0 0 0) are as small as 0.01 cm$${}^{-1}$$, which is remarkable. Moreover, the EE CFA description for the symmetric-stretch fundamental, (1 0 0), is nearly as accurate as for (0 0 0)—reflecting the aforementioned vanishing Coriolis coupling for this motion. As a rule, however, excitations lead to rapid growth of the errors, with the EE error for $$(0\ 1\ 0)$$ increasing to 0.22 cm$${}^{-1}$$, and that of $$J=10$$ (0 0 0) around 1.25 cm$${}^{-1}$$. In contrast, VBE errors for $$J=1$$ (0 0 0) are around 0.06 cm$${}^{-1}$$, and for most rovibrationally excited states are significantly *smaller* than EE errors, especially for the bending excitations.

**Table 2:** $$J=1$$ rovibrational energy levels of H$${}_{2}$$$${}^{16}$$O using the exact Hamiltonian, $$ {\hat{H}} $$, the Coriolis-free Hamiltonian, $$ {\hat{H}} -{\widehat{T}}_{{\rm{V}}{\rm{R}}}$$, and the diagonal $${{\bf{G}}}_{{\rm{R}}}$$ approximation. The results are provided in cm$${}^{-1}$$ and correspond to valence bisector (VBE), Radau bisector (RBE), and Eckart (EE) embeddings, and they are given relative to the appropriate vibrational parent state. Vibrational (vib $${v}_{1}\ {v}_{2}\ {v}_{3}$$) and rotational (rot, $${J}_{{K}_{a}{K}_{c}}$$) quantum numbers are assigned for each rovibrational state. The differences of the eigenvalues obtained with the full and the Coriolis-free [$$\Delta ({\widehat{T}}_{{\rm{V}}{\rm{R}}})$$], and with the Coriolis-free and the diagonal $${{\bf{G}}}_{{\rm{R}}}$$ operators [$$\Delta $$($${G}_{xy}$$)] are also provided.

${ {\hat{H}} }_{{\bf{V}}{\bf{R}}}$				${ {\hat{H}} }_{{\bf{V}}{\bf{R}}}-{\widehat{T}}_{{\bf{V}}{\bf{R}}}$			diagonal ${{\bf{G}}}_{{\boldsymbol{R}}}$			${\boldsymbol{\Delta }}({\widehat{{\boldsymbol{T}}}}_{{\bf{V}}{\bf{R}}})$			${\boldsymbol{\Delta }}$(${{\boldsymbol{G}}}_{{\boldsymbol{x}}{\boldsymbol{y}}}$)		
#	level	vib	rot	VBE	RBE	EE	VBE	RBE	EE	VBE	RBE	EE	VBE	RBE	EE
1	23.8	(0 0 0)	${1}_{01}$	23.9	23.8	23.8	23.9	23.8	23.8	0.07	0.03	0.01	0.00	0.00	0.00
2	37.1	(0 0 0)	${1}_{11}$	37.2	37.2	37.2	37.2	37.2	37.2	0.05	0.03	0.01	0.00	0.00	0.00
3	42.4	(0 0 0)	${1}_{10}$	42.4	42.4	42.4	42.4	42.4	42.4	0.00	0.00	0.00	0.00	0.00	0.00
4	23.8	(0 1 0)	${1}_{01}$	24.0	23.9	24.0	24.0	23.9	24.0	0.16	0.12	0.22	0.00	0.00	0.00
5	40.2	(0 1 0)	${1}_{11}$	40.4	40.3	40.4	40.4	40.3	40.4	0.14	0.12	0.21	0.00	0.00	0.00
6	45.8	(0 1 0)	${1}_{10}$	45.8	45.8	45.8	45.8	45.8	45.8	0.00	0.00	0.00	0.00	0.00	0.00
7	23.8	(0 2 0)	${1}_{01}$	24.1	24.0	24.2	24.1	24.0	24.2	0.24	0.20	0.43	0.00	0.00	0.00
8	44.5	(0 2 0)	${1}_{11}$	44.7	44.7	44.9	44.7	44.7	44.9	0.22	0.21	0.41	0.00	0.00	0.00
9	50.3	(0 2 0)	${1}_{10}$	50.3	50.3	50.3	50.3	50.3	50.3	0.00	0.00	0.00	0.00	0.00	0.00
10	23.4	(1 0 0)	${1}_{01}$	23.5	23.4	23.4	23.5	23.4	23.4	0.07	0.03	0.01	0.00	0.00	0.00
11	36.2	(1 0 0)	${1}_{11}$	36.3	36.3	36.3	36.3	36.3	36.3	0.05	0.03	0.01	0.00	0.00	0.00
12	41.4	(1 0 0)	${1}_{10}$	41.4	41.4	41.4	41.4	41.4	41.4	0.00	0.00	0.00	0.00	0.00	0.00
13	23.6	(0 0 1)	${1}_{01}$	23.6	23.6	23.4	23.6	23.6	23.4	0.06	$-0.01$	$-0.16$	0.00	0.00	0.00
14	35.8	(0 0 1)	${1}_{11}$	35.8	35.8	35.6	35.8	35.8	35.6	$-0.01$	$-0.01$	$-0.16$	0.00	0.00	0.00
15	41.1	(0 0 1)	${1}_{10}$	41.1	41.1	41.1	41.1	41.1	41.1	0.00	0.00	0.00	0.00	0.00	0.00
16	23.8	(0 3 0)	${1}_{01}$	24.1	24.1	24.4	24.1	24.1	24.4	0.32	0.29	0.65	0.00	0.00	0.00
17	50.7	(0 3 0)	${1}_{11}$	51.0	51.0	51.3	51.0	51.0	51.3	0.31	0.29	0.62	0.00	0.00	0.00
18	56.8	(0 3 0)	${1}_{10}$	56.8	56.8	56.8	56.8	56.8	56.8	0.00	0.00	0.00	0.00	0.00	0.00
19	23.4	(1 1 0)	${1}_{01}$	23.6	23.5	23.6	23.6	23.6	23.6	0.16	0.12	0.22	0.00	0.00	0.00
20	39.2	(1 1 0)	${1}_{11}$	39.3	39.3	39.4	39.3	39.3	39.4	0.14	0.12	0.21	0.00	0.00	0.00
21	44.7	(1 1 0)	${1}_{10}$	44.7	44.7	44.7	44.7	44.7	44.7	0.00	0.00	0.00	0.00	0.00	0.00
22	23.6	(0 1 1)	${1}_{01}$	23.7	23.7	23.6	23.7	23.7	23.6	0.14	0.07	0.04	0.00	0.00	0.00
23	38.5	(0 1 1)	${1}_{11}$	38.6	38.6	38.5	38.6	38.6	38.5	0.08	0.07	0.03	0.00	0.00	0.00
24	44.1	(0 1 1)	${1}_{10}$	44.1	44.1	44.1	44.1	44.1	44.1	0.00	0.00	0.00	0.00	0.00	0.00
25	23.7	(0 4 0)	${1}_{01}$	24.1	24.1	24.6	24.1	24.1	24.6	0.40	0.36	0.90	0.00	0.00	0.00
26	60.8	(0 4 0)	${1}_{11}$	61.2	61.2	61.6	61.2	61.2	61.6	0.39	0.37	0.83	0.00	0.00	0.00
27	67.1	(0 4 0)	${1}_{10}$	67.1	67.1	67.1	67.1	67.1	67.1	0.00	0.00	0.00	0.00	0.00	0.00
28	23.4	(1 2 0)	${1}_{01}$	23.7	23.6	23.9	23.7	23.6	23.9	0.26	0.22	0.43	0.00	0.00	0.00
29	43.2	(1 2 0)	${1}_{11}$	43.5	43.4	43.6	43.5	43.4	43.6	0.23	0.21	0.41	0.00	0.00	0.00
30	49.0	(1 2 0)	${1}_{10}$	49.0	49.0	49.0	49.0	49.0	49.0	0.00	0.00	0.00	0.00	0.00	0.00
31	23.6	(0 2 1)	${1}_{01}$	23.9	23.8	23.9	23.9	23.8	23.9	0.23	0.16	0.25	0.00	0.00	0.00
32	42.2	(0 2 1)	${1}_{11}$	42.3	42.3	42.4	42.3	42.3	42.4	0.16	0.16	0.21	0.00	0.00	0.00
33	48.1	(0 2 1)	${1}_{10}$	48.1	48.1	48.1	48.1	48.1	48.1	0.00	0.00	0.00	0.00	0.00	0.00
34	23.0	(2 0 0)	${1}_{01}$	23.1	23.1	23.0	23.1	23.1	23.0	0.08	0.03	$-0.02$	$-0.01$	$-0.01$	$-0.01$
35	35.3	(2 0 0)	${1}_{11}$	35.3	35.3	35.2	35.3	35.3	35.3	0.03	0.02	$-0.03$	$-0.01$	$-0.01$	$-0.01$
36	40.5	(2 0 0)	${1}_{10}$	40.5	40.5	40.5	40.5	40.5	40.5	0.00	0.00	0.00	0.00	0.00	0.00
37	23.2	(1 0 1)	${1}_{01}$	23.3	23.2	23.0	23.2	23.2	23.0	0.07	0.00	$-0.16$	0.01	0.01	0.01
38	34.9	(1 0 1)	${1}_{11}$	34.9	34.9	34.8	34.9	34.9	34.8	$-0.01$	$-0.01$	$-0.16$	0.01	0.01	0.01
39	40.2	(1 0 1)	${1}_{10}$	40.2	40.2	40.2	40.2	40.2	40.2	0.00	0.00	0.00	0.00	0.00	0.00
40	23.3	(0 0 2)	${1}_{01}$	23.3	23.2	23.0	23.3	23.2	23.0	0.04	$-0.05$	$-0.30$	0.00	0.00	0.00
41	34.6	(0 0 2)	${1}_{11}$	34.5	34.5	34.3	34.5	34.5	34.3	$-0.05$	$-0.05$	$-0.27$	0.00	0.00	0.00
42	39.9	(0 0 2)	${1}_{10}$	39.9	39.9	39.9	39.9	39.9	39.9	0.00	0.00	0.00	0.00	0.00	0.00
43	23.6	(0 5 0)	${1}_{01}$	24.1	24.1	24.8	24.1	24.1	24.8	0.46	0.43	1.17	0.00	0.00	$-0.01$
44	80.3	(0 5 0)	${1}_{11}$	80.7	80.7	81.3	80.7	80.7	81.3	0.46	0.44	1.05	0.00	0.00	$-0.01$
45	86.7	(0 5 0)	${1}_{10}$	86.7	86.7	86.7	86.7	86.7	86.7	0.00	0.00	0.00	0.00	0.00	0.00
46	23.4	(1 3 0)	${1}_{01}$	23.8	23.7	24.1	23.8	23.7	24.1	0.35	0.31	0.67	0.00	0.00	0.00
47	49.3	(1 3 0)	${1}_{11}$	49.7	49.6	49.9	49.7	49.6	50.0	0.32	0.30	0.62	0.00	0.00	−0.01
48	55.4	(1 3 0)	${1}_{10}$	55.4	55.4	55.4	55.4	55.4	55.4	0.00	0.00	0.00	0.00	0.00	0.00
49	23.6	(0 3 1)	${1}_{01}$	23.9	23.9	24.1	23.9	23.9	24.1	0.31	0.24	0.48	0.00	0.00	0.00
50	47.4	(0 3 1)	${1}_{11}$	47.6	47.6	47.8	47.6	47.6	47.8	0.24	0.24	0.39	0.00	0.00	0.00
51	53.5	(0 3 1)	${1}_{10}$	53.5	53.5	53.5	53.5	53.5	53.5	0.00	0.00	0.00	0.00	0.00	0.00

**Table 3:** $$J=10$$ rovibrational energy levels of the H$${}_{2}$$$${}^{16}$$O using the exact Hamiltonian, $$ {\hat{H}} $$, and the Coriolis-free Hamiltonian, $$ {\hat{H}} -{\widehat{T}}_{{\rm{V}}{\rm{R}}}$$. The results are provided in cm$${}^{-1}$$ and correspond to valence bisector (VBE), Radau bisector (RBE), and Eckart (EE) embeddings, and they are given relative to the vibrational parent state. Vibrational ($${v}_{1}\ {v}_{2}\ {v}_{3}$$) and rotational (rot, $${J}_{{K}_{a},{K}_{c}}$$) quantum numbers are assigned for each state. The differences of the eigenvalues obtained with the full and the Coriolis-free operators [$$\Delta ({\widehat{T}}_{{\rm{V}}{\rm{R}}})$$] are also provided.

rot	(0 0 0)				(0 1 0)				(0 2 0)				(1 0 0)				(0 0 1)			
	${ {\hat{H}} }_{{\bf{V}}{\bf{R}}}$	${\boldsymbol{\Delta }}({\widehat{{\boldsymbol{T}}}}_{{\bf{V}}{\bf{R}}})$			${ {\hat{H}} }_{{\bf{V}}{\bf{R}}}$	${\boldsymbol{\Delta }}({\widehat{{\boldsymbol{T}}}}_{{\bf{V}}{\bf{R}}})$			${ {\hat{H}} }_{{\bf{V}}{\bf{R}}}$	${\boldsymbol{\Delta }}({\widehat{{\boldsymbol{T}}}}_{{\bf{V}}{\bf{R}}})$			${ {\hat{H}} }_{{\bf{V}}{\bf{R}}}$	${\boldsymbol{\Delta }}({\widehat{{\boldsymbol{T}}}}_{{\bf{V}}{\bf{R}}})$			${ {\hat{H}} }_{{\bf{V}}{\bf{R}}}$	${\boldsymbol{\Delta }}({\widehat{{\boldsymbol{T}}}}_{{\bf{V}}{\bf{R}}})$		
		VBE	RBE	EE		VBE	RBE	EE		VBE	RBE	EE		VBE	RBE	EE		VBE	RBE	EE
$1{0}_{0,10}$	1114.6	5.85	2.56	1.24	1110.5	14.25	11.13	20.47	1108.9	22.30	19.36	39.85	1093.4	6.03	2.79	1.21	1096.9	2.55	$-1.38$	$-15.66$
$1{0}_{1,10}$	1114.6	5.85	2.56	1.24	1110.5	14.25	11.13	20.48	1109.0	22.32	19.38	39.88	1093.4	6.03	2.78	1.22	1096.9	2.55	$-1.38$	$-15.67$
$1{0}_{1,9}$	1293.1	4.55	1.88	0.88	1308.5	10.55	8.03	14.90	1328.9	15.92	13.56	28.21	1268.8	4.70	2.07	0.87	1271.4	2.41	$-0.75$	$-11.99$
$1{0}_{2,9}$	1293.7	4.57	1.90	0.88	1309.8	10.67	8.15	15.05	1331.8	16.31	13.94	28.82	1269.4	4.71	2.07	0.88	1271.7	2.43	7.00	$-12.02$
$1{0}_{2,8}$	1438.1	3.35	1.27	0.68	1463.8	7.05	5.10	9.65	1492.8	9.93	8.12	17.22	1412.2	3.50	1.43	0.68	1415.3	2.48	$-0.71$	$-8.18$
$1{0}_{3,8}$	1446.2	3.53	1.42	0.69	1478.1	7.95	5.95	10.87	1518.3	12.13	10.25	20.71	1419.3	3.61	1.52	0.72	1420.2	2.25	$-0.78$	$-8.67$
$1{0}_{3,7}$	1538.3	2.33	0.73	0.54	1567.7	4.48	2.95	5.73	1601.3	6.57	5.11	10.84	1512.1	2.47	0.85	0.55	1517.9	2.83	$-0.30$	$-4.26$
$1{0}_{4,7}$	1581.4	2.79	1.14	0.59	1630.0	6.30	4.74	8.25	1690.7	9.95	8.47	16.03	1550.9	2.84	1.21	0.49	1549.0	1.90	$-0.56$	$-6.05$
$1{0}_{4,6}$	1616.6	2.03	0.67	0.49	1659.1	4.39	3.05	5.41	1713.0	7.47	6.15	11.70	1589.8	3.05	1.86	−2.12	1599.5	3.09	0.66	$-1.33$
$1{0}_{5,6}$	1718.8	2.32	1.05	0.55	1788.7	5.56	4.35	7.02	1875.7	9.14	7.99	13.84	1678.1	1.91	0.22	−1.23	1678.7	1.54	$-0.37$	$-4.82$
$1{0}_{5,5}$	1724.8	2.13	0.93	0.52	1792.8	5.17	4.01	6.44	1878.4	8.71	7.59	13.19	1694.4	2.40	1.03	0.22	1686.3	1.69	$-0.30$	$-3.77$
$1{0}_{6,5}$	1875.0	1.93	1.04	0.58	1970.1	5.01	4.17	6.29	2087.1	8.36	7.58	12.27	1840.0	2.19	1.13	0.44	1825.0	1.00	$-0.20$	$-4.07$
$1{0}_{6,4}$	1875.5	1.91	1.02	0.57	1970.4	4.97	4.14	6.24	2086.1	7.53	6.53	14.82	1840.2	2.12	1.10	0.43	1825.7	1.02	$-0.19$	$-3.95$
$1{0}_{7,4}$	2054.4	1.45	0.97	0.65	2176.1	4.22	3.79	5.48	2322.5	7.28	6.90	10.53	2013.6	1.55	1.00	0.64	1992.8	0.43	0.09	$-3.17$
$1{0}_{7,3}$	2054.4	1.45	0.97	0.65	2176.1	4.22	3.79	5.48	2322.5	7.27	6.90	10.53	2013.6	1.55	1.00	0.64	1992.8	0.43	0.09	$-3.16$
$1{0}_{8,3}$	2254.3	0.84	0.81	0.76	2402.9	3.11	3.12	4.48	2577.4	5.43	5.48	8.28	2208.5	0.89	0.84	0.91	2179.9	−0.64	0.00	$-1.68$
$1{0}_{8,2}$	2254.3	0.84	0.81	0.76	2402.9	3.11	3.12	4.48	2577.4	5.42	5.48	8.28	2208.5	0.89	0.83	0.91	2179.9	−0.64	0.00	$-1.68$
$1{0}_{9,2}$	2471.2	0.11	0.57	0.88	2646.3	1.67	2.15	3.28	2845.4	3.21	3.71	5.26	2422.8	0.29	0.74	1.38	2383.3	−1.64	0.09	0.08
$1{0}_{9,1}$	2471.2	0.11	0.57	0.88	2646.3	1.67	2.15	3.28	2845.4	3.21	3.71	5.26	2422.8	0.29	0.74	1.38	2383.3	−1.64	0.09	0.08
$1{0}_{10,1}$	2701.8	$-0.73$	0.23	0.99	2902.5	$-0.12$	0.84	1.87	3113.5	$-0.06$	0.90	1.80	2661.7	$-0.15$	0.81	2.02	2599.7	$-2.80$	0.11	2.25
$1{0}_{10,0}$	2701.8	$-0.73$	0.23	0.99	2902.5	$-0.12$	0.84	1.87	3113.5	$-0.06$	0.90	1.80	2661.7	$-0.15$	0.81	2.02	2599.7	$-2.80$	0.11	2.25

In Table 1, explicit ${{\bf{G}}}_{{\rm{V}}{\rm{R}}}$ and ${{\bf{G}}}_{{\rm{R}}}$ tensor elements are provided for the Eckart embedding, for both valence and Radau vibrational coordinates, at several different geometries. The ${{\bf{G}}}_{{\rm{V}}{\rm{R}}}$/${{\bf{G}}}_{{\rm{R}}}$ tensor elements are seen to be vibrational coordinate dependent/independent, as expected. Table 1 also lists values for the Frobenius norm of $({{\bf{G}}}_{{\rm{R}}}-{{\bf{g}}}_{{\rm{R}}}^{-1})$ (*i.e*., $| | {{\bf{G}}}_{{\rm{R}}}-{{\bf{g}}}_{{\rm{R}}}^{-1}| {| }_{{\rm{F}}}$), which are independent of the choice of vibrational coordinates. The matrix elements for RBE and VBE are also shown. In order to gain a deeper understanding of the manifestation of CC in the studied embeddings, it is worth comparing $| | {{\bf{G}}}_{{\rm{R}}}-{{\bf{g}}}_{{\rm{R}}}^{-1}| {| }_{{\rm{F}}}$ values at different geometries; again, this is taken as a measure of the extent of CC.

For the reference geometry, VBE shows nonzero CC, whereas both RBE and EE show zero CC, as expected. On the other hand, RBE exhibits zero CC across *all* ${C}_{2v}$ distortions, a considerable advantage of this embedding. This is unlike EE, which has substantially larger CC, even compared to VBE, when there is bending excitation, although for pure symmetric stretching EE has zero CC, as well. For ${C}_{s}$ geometries, either RBE or EE can be better than the other embedding, depending on the actual distortion. For pure asymmetric stretch distortions, EE is better; however, the EE $| | {{\bf{G}}}_{{\rm{R}}}-{{\bf{g}}}_{{\rm{R}}}^{-1}| {| }_{{\rm{F}}}$ values increase rapidly for combinations with any bend excitation. In contrast, RBE values remain more or less constant with increasing bend excitation, reflecting the fact that bending is a symmetric motion. Note that *most* geometries of Table 1 involve a combination of “excitations”; hence, one can surmise that RBE CC overall is smaller. As to the VBE $| | {{\bf{G}}}_{{\rm{R}}}-{{\bf{g}}}_{{\rm{R}}}^{-1}| {| }_{{\rm{F}}}$ values, we observe that these are only slightly larger than RBE for the ${C}_{s}$ geometries considered.

Of course, the “acid test” for a given embedding is the accuracy of its CFA rovibrational energy levels. Based on $| | {{\bf{G}}}_{{\rm{R}}}-{{\bf{g}}}_{{\rm{R}}}^{-1}| {| }_{{\rm{F}}}$ values, RBE is expected to be the most accurate choice among the three embeddings considered here—particularly for symmetric stretch and bend excitations. Indeed, this is the case. As Table 2 and Fig. 3 attest, the $J=1$ RBE errors can be almost as small as EE errors for those few cases where EE is the best, yet significantly smaller than EE and VBE errors in all other cases. Even for pure asymmetric stretch excitations, RBE errors are about an order of magnitude *smaller* than EE, as seen from Table 2. For higher $J$, this trend is even more evident (see Fig. 4).

Overall, all three linear embedding CFA results reproduce the exact values remarkably well. For the lowest 50 rovibrational states, the largest errors are about 1 cm${}^{-1}$ for $J=1$, and 20 cm${}^{-1}$ for $J=10$. Additionally, for $J=1$—for every single vibrational state and for every embedding—the Coriolis-free prediction for the ${1}_{01}$ state rovibrational energy is *exact*. This remarkable finding relates to parity, as explained in the next subsection.

### The diagonal G${}_{{\bf{R}}}$ approximation (DGRA)

The next rung in our descending hierarchy is the diagonal **G**${}_{{\rm{R}}}$ approximation. For all ${C}_{2v}$ geometries (including the reference geometry), ${G}_{xy}^{{\rm{R}}}=0$. Only for large asymmetric displacements do we expect ${G}_{xy}^{{\rm{R}}}$ to become substantial; accordingly, only for excited asymmetric stretch states do we expect to see a large difference from CFA. In actuality, however, the DGRA energy levels are *extremely* close to their CFA counterparts—much more so than might be expected (see Table 2). Most discrepancies are substantially smaller—and none are significantly larger—than 0.01 cm${}^{-1}$, across the full range of vibrational excitations considered. We also note that for *all* embeddings the computed ${1}_{01}$ DGRA levels are still exact. This is because ${G}_{xy}^{{\rm{R}}}$ does not contribute to the $1\times 1$ negative-parity block. As another important conclusion of the present study, from a practical standpoint, *there is no reason not to use the diagonal* ${{\bf{G}}}_{{\rm{R}}}$ *approximation*, if one is committed to throwing away Coriolis coupling anyway (at least for linear embeddings).

DGRA introduces a new permutation symmetry, which can be very useful in practice. Note that permutation affects rotation and vibration *simultaneously*. In the vibrational space, permutation changes the sign of the asymmetric stretch displacement, without affecting the symmetric displacements. In the rotational space, even-$\bar{K}$ corresponds to one $\varepsilon$ value, and odd-$\bar{K}$ to the other. The effect of permutation on the two spaces is coupled solely through the ${G}_{xy}^{{\rm{R}}}$ contribution. Consequently, without ${G}_{xy}^{{\rm{R}}}$, one obtains *two independent permutation symmetries*—a *vibrational* permutation symmetry ${\varepsilon }_{{\rm{v}}{\rm{i}}{\rm{b}}}$, and an independent *rotational* permutation symmetry ${\varepsilon }_{{\rm{r}}{\rm{o}}{\rm{t}}}$. It may be feasible to utilize this extra information during the assignment of rovibrational states.

As a practical benefit, this additional symmetry allows a further two-fold reduction in the basis size, taking us up to $n=8$. Furthermore, because DGRA is so close to CFA—which in turn does an excellent job of modeling the exact rovibrational energies—both ${\varepsilon }_{{\rm{v}}{\rm{i}}{\rm{b}}}$ and ${\varepsilon }_{{\rm{r}}{\rm{o}}{\rm{t}}}$ are *nearly perfectly* good quantum numbers. Indeed, the usual association for AB${}_{2}$ molecules of even-${v}_{3}$ vibrational quantum states with even permutation symmetry, and odd-${v}_{3}$ states with odd permutation symmetry, is in fact a manifestation of ${\varepsilon }_{{\rm{v}}{\rm{i}}{\rm{b}}}$, rather than $\varepsilon$ itself.

### The generalized CS approximation (GCSA)

The CS approximation can *drastically* reduce the computational cost, especially for large $J$. The idea is very simple: set all off-diagonal $K$ blocks in ${\widetilde{H}}^{J}$ equal to zero. $K$ then becomes a good quantum number—thus, once again, increasing the symmetry of the Hamiltonian. Since the remaining diagonal $K={K}^{{\prime} }$ blocks can be diagonalized separately, the basis size of the problem reduces by a factor of $n=(2J+1)$. For $J > 3$, this provides a greater computational reduction than the DGRA, for which $n=8$. Actually, there are some modest savings for $J=2$ and $J=3$, as well, for which not all of the $n=8$ irreps are realized in the diagonal ${{\bf{G}}}_{{\rm{R}}}$ case.

The discarded $K\ne {K}^{{\prime} }$ blocks are often loosely referred to as “Coriolis coupling”, although they are clearly not ${\widetilde{T}}_{{\rm{V}}{\rm{R}}}$. In general, what these off-diagonal blocks represent depends on the embedding, as well as the particular body-fixed axis, ${\hat{c}}$, along which $K$ is projected. It is convenient to choose ${\hat{c}}$ to correspond to $\widehat{x}$, ${\hat{y}}$, or ${\hat{z}}$, although strictly speaking any (global) orientation may be used. For planar molecules, the choice ${\hat{c}}= {\hat{z}}$ may be expected to be particularly poor. All three choices, ${\hat{c}}=\widehat{x}$, ${\hat{c}}={\hat{y}}$, and ${\hat{c}}= {\hat{z}}$, are considered here.

Previous applications of the CS approximation have been in the context of Jacobi and Radau vector embeddings, for which ${\widetilde{H}}^{J}$ is tridiagonal. In the present, *generalized* CS context, we are also discarding a block-pentadiagonal contribution, which may in principle be quite large. Note that GCSA is equivalent to the following prescription:

1. Start with the DGRA matrix representation, and identify the projection axis ${\hat{c}}$ and the diagonal tensor elements ${G}_{aa}^{{\rm{R}}}$ and ${G}_{bb}^{{\rm{R}}}$.
2. Replace both ${G}_{aa}^{{\rm{R}}}$ and ${G}_{bb}^{{\rm{R}}}$ with the *average* value $({G}_{aa}^{{\rm{R}}}+{G}_{bb}^{{\rm{R}}})/2$.

Step 2 automatically results in a *symmetric* rotor form. It is interesting that this prescription seems to answer an “age-old” debate about how best to go from an asymmetric to a symmetric rotor form^39^: should one average two moments of inertia or two rotational constants? Evidently, the latter is the more correct approach.

GCSA results are presented for $J=1$ in Table 4, for each of the three choices of ${\hat{c}}$ (*i.e*., CS${}_{x}$, CS${}_{y}$, and CS${}_{z}$), for the RBE (results for the other linear embeddings are similar). Note that $J=1$ is special, in that each GCSA calculation results in one *exact* diagonal ${{\bf{G}}}_{{\rm{R}}}$ eigenvalue per vibrational parent. Although CS${}_{z}$ follows the trend of the former approximations, by averaging ${G}_{xx}^{{\rm{R}}}$ and ${G}_{yy}^{{\rm{R}}}$ the ${1}_{10}$ levels remain unchanged, and CS${}_{x}$ yields the smallest errors. The errors are on the order of $\pm$2.5 cm${}^{-1}$ for the zero-point vibration, compared to $\pm$6.7 cm${}^{-1}$ and $\pm$9.3 cm${}^{-1}$ for CS${}_{z}$ and CS${}_{y}$. Surprisingly, CS${}_{x}$ errors do not increase appreciably with vibrational excitation.

**Table 4:** $J=1$ rovibrational energy levels of H${}_{2}$^16^O in Radau bisector embedding with different generalized centrifugal sudden approximations (GCSA) referring to each of the three choices of ${\hat{c}}$ axis along which $K$ is projected. The results are provided in cm${}^{-1}$ and given relative to the vibrational parent state. Vibrational [vib, $({v}_{1}\ {v}_{2}\ {v}_{3})$] and rotational (rot, ${J}_{{K}_{a}{K}_{c}}$) quantum numbers are assigned for each state. The differences of the eigenvalues compared to the diagonal ${{\bf{G}}}_{{\rm{R}}}$ approximation (DGRA) [$\Delta$(GCSA)] are also provided.

${ {\hat{H}} }_{{\rm{V}}{\rm{R}}}$				DGRA	GCSA			$\Delta$(GCSA)		
#	level	vib	rot	ref.	CS${}_{{\bf{x}}}$	CS${}_{{\bf{z}}}$	CS${}_{{\bf{y}}}$	CS${}_{{\bf{x}}}$	CS${}_{{\bf{z}}}$	CS${}_{{\bf{y}}}$
1	23.8	(0 0 0)	${1}_{01}$	23.8	23.8	30.5	33.1	0.0	6.7	9.3
2	37.1	(0 0 0)	${1}_{11}$	37.2	39.8	30.5	37.2	2.6	$-6.7$	0.0
3	42.4	(0 0 0)	${1}_{10}$	42.4	39.8	42.4	33.1	$-2.6$	0.0	$-9.3$
4	23.8	(0 1 0)	${1}_{01}$	23.9	23.9	32.3	34.9	0.0	8.3	10.9
5	40.2	(0 1 0)	${1}_{11}$	40.3	43.1	32.3	40.3	2.7	$-8.1$	0.0
6	45.8	(0 1 0)	${1}_{10}$	45.8	43.1	45.8	34.9	$-2.7$	0.0	$-10.9$
7	23.8	(0 2 0)	${1}_{01}$	24.0	24.0	34.6	37.2	0.0	10.6	13.2
8	44.5	(0 2 0)	${1}_{11}$	44.7	47.5	34.6	44.7	2.8	$-10.1$	0.0
9	50.3	(0 2 0)	${1}_{10}$	50.3	47.5	50.3	37.2	$-2.8$	0.0	$-13.1$
10	23.4	(1 0 0)	${1}_{01}$	23.4	23.4	29.8	32.4	0.0	6.4	9.0
11	36.2	(1 0 0)	${1}_{11}$	36.3	38.9	29.8	36.3	2.6	$-6.4$	0.0
12	41.4	(1 0 0)	${1}_{10}$	41.4	38.9	41.4	32.4	$-2.6$	0.0	$-9.0$
13	23.6	(0 0 1)	${1}_{01}$	23.6	23.6	29.5	32.3	0.0	6.0	8.8
14	35.8	(0 0 1)	${1}_{11}$	35.8	38.4	29.5	35.8	2.6	$-6.2$	0.0
15	41.1	(0 0 1)	${1}_{10}$	41.1	38.4	41.1	32.3	$-2.6$	0.0	$-8.7$
16	23.8	(0 3 0)	${1}_{01}$	24.1	24.1	38.0	40.5	0.0	13.9	16.4
17	50.7	(0 3 0)	${1}_{11}$	51.0	53.9	38.0	51.0	2.9	$-13.0$	0.0
18	56.8	(0 3 0)	${1}_{10}$	56.8	53.9	56.8	40.5	$-2.9$	0.0	$-16.3$
19	23.4	(1 1 0)	${1}_{01}$	23.6	23.6	31.5	34.1	0.0	8.0	10.6
20	39.2	(1 1 0)	${1}_{11}$	39.3	42.0	31.5	39.3	2.7	$-7.8$	0.0
21	44.7	(1 1 0)	${1}_{10}$	44.7	42.0	44.7	34.1	$-2.7$	0.0	$-10.6$
22	23.6	(0 1 1)	${1}_{01}$	23.7	23.7	31.1	33.9	0.0	7.4	10.2
23	38.5	(0 1 1)	${1}_{11}$	38.6	41.3	31.1	38.6	2.8	$-7.5$	0.0
24	44.1	(0 1 1)	${1}_{10}$	44.1	41.3	44.1	33.9	$-2.8$	0.0	$-10.2$
25	23.7	(0 4 0)	${1}_{01}$	24.1	24.1	43.3	45.8	0.0	19.2	21.7
26	60.8	(0 4 0)	${1}_{11}$	61.2	64.1	43.3	61.2	3.0	$-17.8$	0.0
27	67.1	(0 4 0)	${1}_{10}$	67.1	64.1	67.1	45.8	$-3.0$	0.0	$-21.3$
28	23.4	(1 2 0)	${1}_{01}$	23.6	23.6	33.8	36.4	0.0	10.1	12.7
29	43.2	(1 2 0)	${1}_{11}$	43.4	46.2	33.8	43.4	2.8	$-9.7$	0.0
30	49.0	(1 2 0)	${1}_{10}$	49.0	46.2	49.0	36.4	$-2.8$	0.0	$-12.7$
31	23.6	(0 2 1)	${1}_{01}$	23.8	23.8	33.2	35.9	0.0	9.4	12.2
32	42.2	(0 2 1)	${1}_{11}$	42.3	45.2	33.2	42.3	2.9	$-9.2$	0.0
33	48.1	(0 2 1)	${1}_{10}$	48.1	45.2	48.1	35.9	$-2.9$	0.0	$-12.1$
34	23.0	(2 0 0)	${1}_{01}$	23.1	23.1	29.1	31.8	0.0	6.1	8.7
35	35.3	(2 0 0)	${1}_{11}$	35.3	37.9	29.1	35.3	2.6	$-6.2$	0.0
36	40.5	(2 0 0)	${1}_{10}$	40.5	37.9	40.5	31.8	$-2.6$	0.0	$-8.7$
37	23.2	(1 0 1)	${1}_{01}$	23.2	23.2	28.9	31.7	0.0	5.7	8.5
38	34.9	(1 0 1)	${1}_{11}$	34.9	37.5	28.9	34.9	2.6	$-6.0$	0.0
39	40.2	(1 0 1)	${1}_{10}$	40.2	37.5	40.2	31.7	$-2.6$	0.0	$-8.5$
40	23.3	(0 0 2)	${1}_{01}$	23.2	23.2	28.7	31.6	0.0	5.4	8.3
41	34.6	(0 0 2)	${1}_{11}$	34.5	37.2	28.7	34.5	2.7	$-5.9$	0.0
42	39.9	(0 0 2)	${1}_{10}$	39.9	37.2	39.9	31.6	$-2.7$	0.0	$-8.3$

## Conclusions

This joint analytical and numerical study offers several interesting and occasionally surprising conclusions about various rotational-vibrational approximations, and associated linear embeddings, employing H$${}_{2}$$$${}^{16}$$O as a canonical test system:

1. The Eckart^51^ embedding is *not* the best choice of embedding, especially when rotational and/or vibrational excitations are significant.
2. There exist embeddings (*e.g*., the Radau bisector embedding) for which Coriolis coupling vanishes over an entire two-dimensional vibrational coordinate subspace (*i.e*., the symmetric vibrational coordinates).
3. The diagonal ${{\bf{G}}}_{{\rm{R}}}$ approximation should be used instead of the Coriolis-free approximation, because the computed energy levels are almost exactly as good but the benefits in terms of symmetry and numerical efficiency are substantial.
4. The diagonal ${{\bf{G}}}_{{\rm{R}}}$ approximation leads to two distinct and almost-perfectly-good parity quantum numbers, one for rotations and one for vibrations.

Note that conclusions (3) and (4) occur only by virtue of permutation symmetry, but should manifest for larger molecules with at least two identical nuclei. As for (2), it is well known that the Eckart embedding achieves zero Coriolis coupling at any given geometry^51^, and that this can be extended over a one-dimensional subspace using “post-Eckart” embeddings, such as the one due to Sayvetz^64^. That two-dimensional subspaces of this kind are also possible, for systems and embeddings where Coriolis coupling as a whole does not vanish, is a novel discovery, so far as we are aware. Even more surprisingly, the Radau bisector embedding that achieves this is *not* Eckart-based.

The full ramifications of these findings certainly merit further investigation, especially vis-à-vis larger and more general molecular systems. In an upcoming paper^41^ we will address other linear and nonlinear embeddings such as those based on Jacobi coordinates, other types of triatomics such as triangular A${}_{3}$ molecules, and also larger molecules. The latter class includes “floppy” systems such as ammonia with an inversion tunneling motion.
